# Molecular size and charge as dimensions to identify and characterize circulating glycoforms of human FSH, LH and TSH

**DOI:** 10.1080/03009734.2017.1412373

**Published:** 2018-01-04

**Authors:** Leif Wide, Karin Eriksson

**Affiliations:** Department of Medical Sciences, Clinical Chemistry, University Hospital, Uppsala University, Sweden

**Keywords:** FSH, glycoforms, glycosylation, LH, sialic acid, sulfonation, TSH

## Abstract

**Background:**

FSH, LH, and TSH are glycoprotein hormones secreted from the pituitary as fully and low-asparagine-glycosylated hormones. These glycoforms of the hormones exist as a large number of isoforms varying in their glycan contents of terminal anionic monosaccharides (AMS), i.e. sialic acid (SA) and sulfonated N-acetylgalactosamine (SU). Due to the immense heterogeneity and the low concentrations in serum it has been a challenge to develop reliable analytical methods to measure and characterize the circulating glycoforms of these hormones.

**Methods:**

The hormones were separated with respect to AMS content per molecule by calibrated 0.1% agarose suspension electrophoreses. Glycoforms in separated fractions were then analyzed with respect to size by 180 calibrated Sephadex G-100 gel filtrations. The hormones were measured with time-resolved sandwich fluoroimmunoassays. All separations and assays were performed in veronal buffer at pH 8.7. Sera and fractions were also analyzed after removal of terminal SA.

**Results:**

In addition to the fully glycosylated FSH, LH, and TSH, also tri-glycosylated FSH and di-glycosylated LH and TSH forms could be identified in serum samples. The low- and fully glycosylated hormones differed both with respect to size and to median number of AMS per molecule. Algorithms, based on the distributions by electrophoreses, were developed for each hormone to estimate percent low-glycosylated forms in serum. The median numbers of SA and SU per glycoform molecule were estimated using results obtained after desialylation.

**Conclusion:**

The methods can be used for identification and characterization of glycoforms of circulating FSH, LH, and TSH in physiological and clinical studies.

## Introduction

Follicle-stimulating hormone (FSH), luteinizing hormone (LH), and thyroid-stimulating hormone (TSH) are glycoprotein hormones with similarities in their chemical structures. They consist of two dissimilar subunits, termed alpha (α) and beta (β), which are joined by non-covalent bonding forming three different heterodimers. The protein part of the α-subunit is identical for the three hormones, while the protein parts of the β-subunits differ. N-glycans (oligosaccharides) can be covalently attached to the proteins at asparagine (Asn) residues by an N-glycosidic bond.

These three hormones are not three single entities. Instead, each hormone exhibits a considerable heterogeneity due to different degrees of glycosylation and differences in glycan composition (1–10). The isoforms can be separated by electrophoresis due to variation in charge determined by their contents of two terminal anionic monosaccharides (AMS): sialic acid (SA) and sulfonated N-acetylgalactosamine (SU). These terminal AMS residues are decisive for the half-lives of the hormones in human blood circulation (11,12).

**Figure 1.**
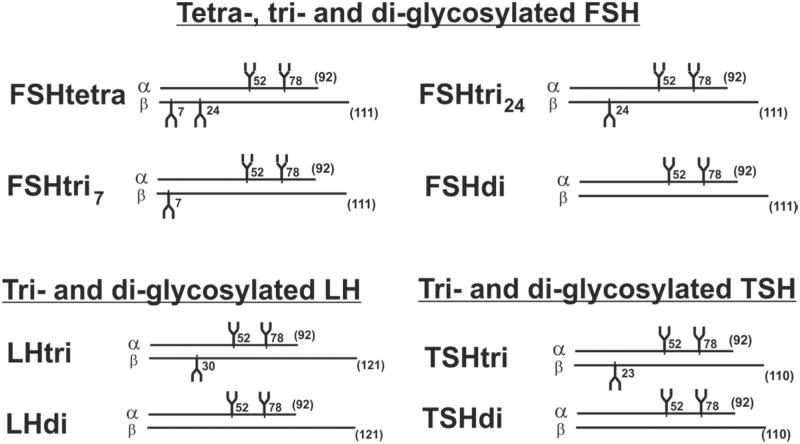
Schematic drawings of structures of the heterodimeric glycoforms of human FSH, LH, and TSH consisting of one α and one β peptide chain. The number of amino acids of the peptide chains is given in parentheses, and the peptide positions of the glycans are indicated.

FSH was first reported by a research group in USA to exist in human pituitaries and in urinary preparations as two major glycoforms designated tetra-glycosylated (FSHtetra) and di-glycosylated FSH (FSHdi) (13,14). Later studies from the same research group showed that a majority of the low-glycosylated FSH was tri- and not di-glycosylated (15,16). We have shown that both FSH and LH exist in serum as low- and fully glycosylated hormones (17). In this study we report that also TSH is present in serum as a low- and fully glycosylated hormone. The α-subunit of these glycoprotein hormones seems always to be decorated with two glycans, at positions 52 and 78 (15,16). The glycoforms of FSH, LH, and TSH with the positions for glycosylation are schematically shown in Figure 1.

Due to the immense heterogeneity and the low concentrations, it is a great challenge to develop reliable analytical methods to measure and characterize circulating glycoforms of FSH, LH, and TSH. The methods described in this report are based on the combination of results from determinations of the number of AMS per molecule by strictly calibrated electrophoreses followed by estimations of molecular size by again strictly calibrated gel filtrations. Measurements after desialylation of the glycoforms with neuraminidase were included. Results have been expressed as the median numbers of AMS, SA, and SU residues per molecule and per glycan for each glycoform of the three hormones in the circulation.

## Methods

### Subjects

Serum samples were collected from different groups of patients and from healthy individuals as previously described (10,17).

### Immunoassay of FSH, LH, and TSH

Concentrations of FSH, LH, and TSH in serum samples and in separated fractions after electrophoreses and gel filtrations were measured using time-resolved sandwich fluoroimmunoassays (Delfia, PerkinElmer-Wallac Oy, Turku, Finland). The methods permitted measurements of the hormones directly in the 0.075 M veronal (Sigma-Aldrich Chemie GmbH, Germany) buffer at pH 8.7 eluted from electrophoreses and gel filtrations. All sera were initially tested to exclude all individuals with the common variant form of LH (18). The detection limit of the three hormones in fractions from electrophoresis was about 100 attogram.

### Neuraminidase treatment

Neuraminidase treatments were performed to remove the terminal SA residues, leaving the SU as the only AMS remaining on the molecules. Serum samples or separated fractions from electrophoresis (500 µL) were desalted and buffer exchanged using an NAP-5 column (GE Healthcare, UK) equilibrated with 10 mL 0.2 M sodium acetate buffer (pH 5.6). Proteins were eluted with 1 mL acetate buffer and then mixed and incubated for 24 h at 37 °C with 70 mU neuraminidase (from *Arthrobacter ureafaciens*, EC 3.2.1.18; MP Biomedicals, Solon, OH, USA). The mixture was then kept at −20 °C until analyzed by electrophoresis. Numbers of SU were determined by the elution pattern after electrophoresis. The number of AMS minus the number of SU represents the number of SA on the molecule.

### Electrophoresis

Electrophoreses were performed at 12–13 °C in columns 1.33 × 65 cm with 0.10% agarose (GE Healthcare, Uppsala, Sweden) suspension in 0.075 M veronal buffer at pH 8.7 for 18 h at a fixed current of 53 mA and a voltage of 1100–1300 (19). The start zone was identified by adding 10 µL of a lipid solution (Intralipid, Fresenius KABI, Uppsala, Sweden) to the sample, and the eluted lipid solution was measured at 340 nm in a spectrophotometer. The zone with albumin was similarly measured at 280 nm. The charge of the eluted components was expressed as electrophoretic mobility in albumin mobility units (AMU) where one AMU is the distance from the start zone to the albumin zone. For each hormone the mobility at the position for molecules without AMS and the increase in mobility per charged group of AMS had been determined for the particular batch of agarose. After electrophoreses the eluted fractions of about 1.3 mL were centrifuged for 45 min at 2400*g* at 12 °C. FSH, LH, and TSH were measured in 250 µL or less of the supernatants. The area of hormones was resolved into peaks at the positions for different number of AMS residues per molecule.

### Gel filtration

**Figure 2.**
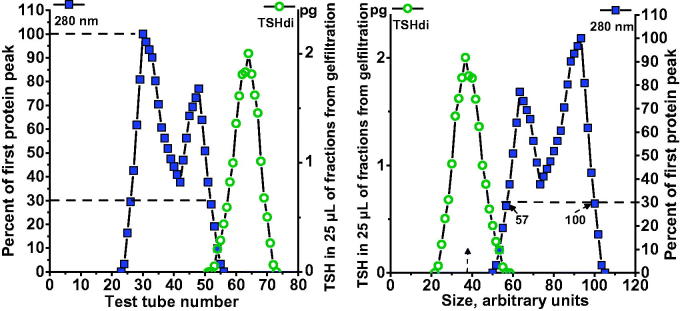
Calibration of Sephadex G-100 gel filtration. The patterns of proteins and hormones in relation to fraction eluted are shown in the *left panel*. The position where the first protein peak, measured at 280 nm, passed 30% of its top level was given an arbitrary value of 100, and the corresponding position on the slope of the albumin peak was given an arbitrary value of 57. The patterns expressed as size in arbitrary units are shown in the *right panel*.

Single fractions from electrophoresis of serum samples were further analyzed by gel filtration. A selected human serum with a low hormone concentration was added to the sample under investigation to form a serum concentration of 17% and then centrifuged for 10 min at 16,000*g*; the final volume added to the column was 800 µL. Gel filtrations were performed in 1.6 × 70 cm columns with Sephadex G-100 (GE Healthcare, Uppsala, Sweden) in 0.075 M veronal buffer at pH 8.7, eluted with a flow rate of 4 mL per h, and each fraction was collected for 12 min. The first eluted protein peaks which included that of albumin, measured at 280 nm, were used as internal standards for calibration of each gel filtration. The position where the first protein peak passed 30% of its top level was given an arbitrary value of 100 (arb.U), and the corresponding position on the slope of the albumin peak was given an arbitrary value of 57 (arb.U), as illustrated in Figure 2. The mean value for serum albumin was 67.5 arb.U.

### Determination of SU and SA on glycoforms

After electrophoreses, the exact positions for the median values of AMS per molecule of the two serum glycoforms of each hormone were determined. Representative aliquots of these fractions for low- and fully glycosylated hormones were neuraminidase-treated and then analyzed by electrophoresis. The number of SU residues and the percentage of SU out of the AMS were determined for each glycoform.

## Results

### Size of glycoforms in serum as estimated by gel filtration

**Figure 3.**
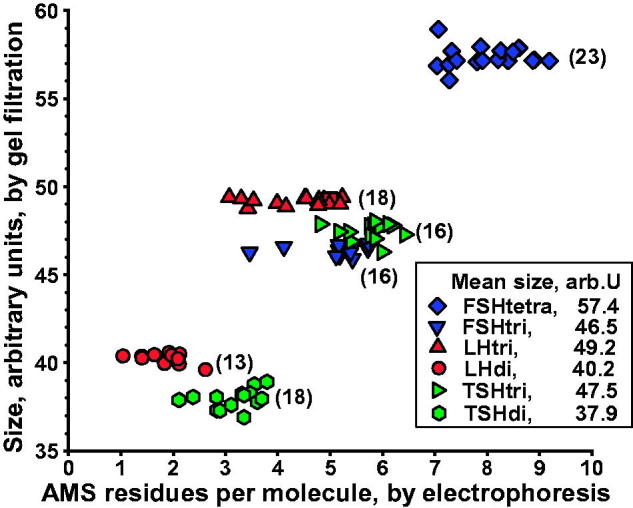
Size of glycoforms of FSH, LH, and TSH in serum, as estimated by gel filtration, in relation to number of anionic monosaccharides (AMS) per molecule, as estimated by electrophoresis. Number of gel filtrations within parentheses. Mean values of glycoform size, in arbitrary units, ranging from 37.9 to 57.4, are shown.

The sizes of different glycoforms in serum, estimated by analyzing single fractions from electrophoresis of a total of 104 gel filtrations, are shown in Figure 3. Fractions from electrophoresis found to include both low- and fully glycosylated hormones were not included. The size values for the low- and fully glycosylated FSH, LH, or TSH fall into distinct groups clearly separated without any overlap. Di-glycosylated FSH and macroforms of fully glycosylated FSH, LH, and TSH, which we detect in pituitary extracts (unpublished observations), were not found in the serum samples.

### Frequencies of low- and fully glycosylated hormones in serum, as estimated by gel filtration

This study included, in addition to those presented above, 76 gel filtrations of fractions from serum electrophoreses at positions containing both low- and fully glycosylated hormone. Based on the results of these gel filtrations, the frequencies of low- versus fully glycosylated hormone were estimated for FSH in six serum samples, LH in five serum samples, and TSH in eight serum samples.

**Figure 4.**
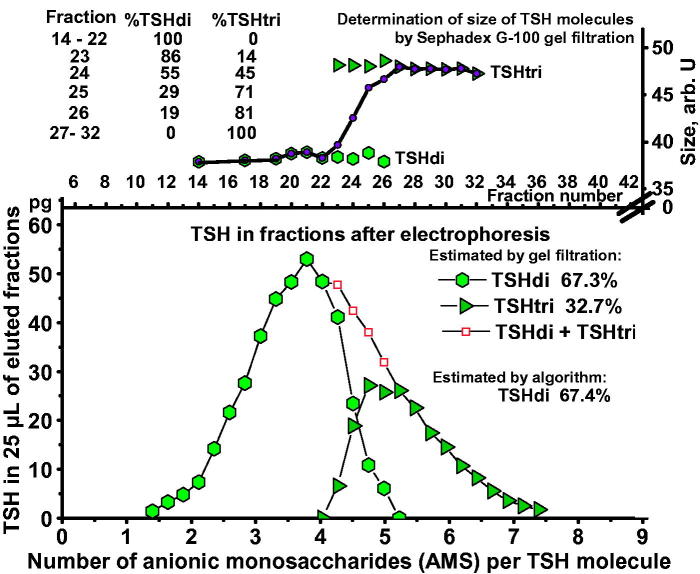
Estimation of percent TSHdi by gel filtration in a serum sample from a 27-year-old woman with hypothyroidism. Distribution of TSH in pg per 25 µL eluate in relation to AMS per molecule by electrophoresis in the *lower panel*. Results of estimations of size of TSH glycoforms in fractions after electrophoresis in the *upper panel*, including measurements where TSHdi and TSHtri overlap.

An example illustrating the method is shown in Figure 4. TSH in a serum sample from a 27-year-old woman with hypothyroidism was first separated by electrophoresis, followed by gel filtrations of fractions 14–32. Fractions 23 to 26 contain both glycoforms with gradually decreasing amounts of TSHdi. When calculated from the results of the gel filtrations the TSH molecules amounted to 67.3% TSHdi and 32.7% TSHtri.

### Algorithms designed to estimate the percent low-glycosylated FSH, LH, and TSH by electrophoresis

**Figure 5.**
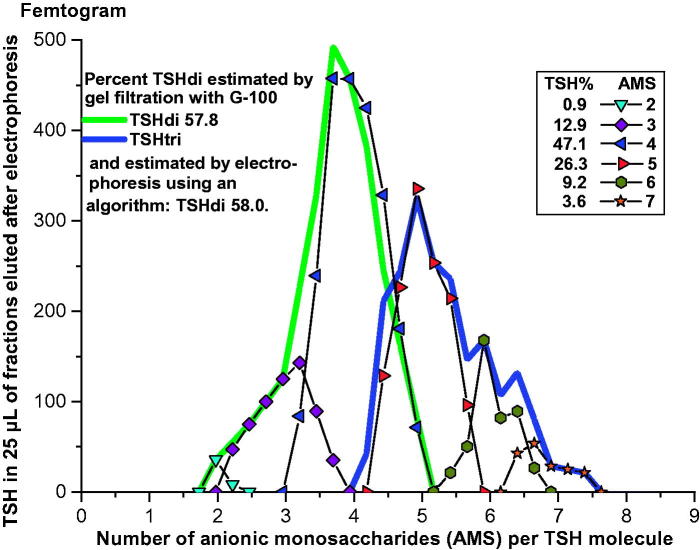
Electrophoresis of TSH in a serum sample from a 34-year-old euthyroid woman. The TSH in femtogram per 25 µL eluate is plotted in peaks in relation to number of AMS per molecule, and the distribution in percent of total amount of TSH eluted is indicated. The distributions of TSHdi and TSHtri from size estimations by gel filtration, and the percent TSHdi estimated by electrophoresis using an algorithm, are shown.

The distribution of the hormone eluted after electrophoresis was expressed in percent at the positions for different numbers of AMS. This is illustrated in Figure 5 where a serum sample from a euthyroid 34-year-old woman was analyzed by electrophoresis. The distribution of TSH in percent at the positions of AMS from 2 to 7 is indicated. The values for the electrophoretic distributions were used in algorithms to calculate the percent low-glycosylated forms. The results were compared with those estimated by gel filtrations, regarded as ‘true’ values. More than 10 different algorithms were investigated for each hormone, and the formulas given below were chosen as being best correlated to the ‘true’ values. In each formula, the letters from ‘a’ to ‘h’ indicate the percent of the hormone eluted at the positions of one to eight AMS per molecule.
(1)
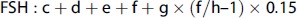

(2)
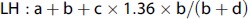

(3)
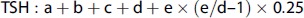



**Figure 6.**
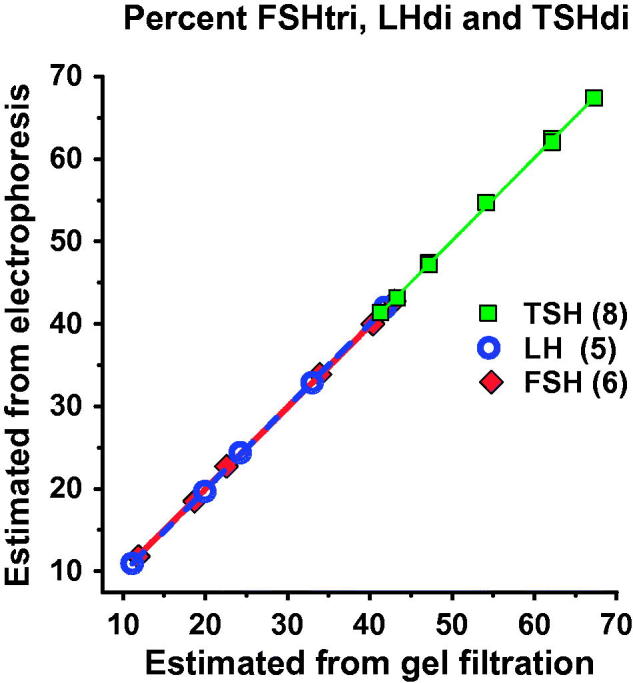
The percent low-glycosylated glycoforms, as estimated by electrophoresis using the three algorithms (see text), was plotted against the corresponding percentage, as estimated by gel filtration. The slopes and the coefficients of correlation were close to 1.

We calculated percent low-glycosylated form of each hormone, as estimated by electrophoresis using the algorithms plotted against the corresponding percentage, as estimated by gel filtration. The slopes and the coefficients of correlation were close to 1 (Figure 6). The median numbers of AMS per molecule and per glycan of low- and fully glycosylated hormones in serum were calculated from the electrophoretic distributions using the algorithms to estimate the frequency of low- versus fully glycosylated hormones. The number of AMS per glycan of the glycoforms of FSH and LH was determined in 240 serum samples and that of TSH in 466 serum samples. The mean numbers of AMS per glycan were for FSHtri 1.94, FSHtetra 1.86, LHdi 1.18, LHtri 1.22, TSHdi 1.89, and TSHtri 1.75. The mean ratios of AMS per glycan on low- versus fully glycosylated hormones were for FSH 1.04, for LH 0.965, and for TSH 1.08.

### Determination of SU and SA on low- and fully glycosylated hormones

The ratio of percent SU out of the AMS per molecule on low- versus fully glycosylated hormone was determined in serum samples from different healthy individuals and patients. The ranges of the ratio values were for FSH 1.25–1.66 (*n* = 25), for LH 1.08–1.44 (*n* = 26), and for TSH 1.19–1.31 (*n* = 21). These ratio values were then used as a factor for calculating the numbers of SU and SA per molecule and per glycan on the low- and fully glycosylated hormones.

## Discussion

The analytical methods described in the present study were developed to measure the concentrations in serum samples of each of the two glycoforms of FSH, LH, and TSH and to determine the median numbers of AMS, SA, and SU per molecule and per glycan for each glycoform. From the distributions after electrophoresis the relative amounts of low- and fully glycosylated forms of each hormone could be estimated by use of the algorithms described.

Each one of the six serum glycoforms, designated FSHtri, FSHtetra, LHdi, LHtri, TSHdi, and TSHtri is heterogeneous and present in circulation as spectra of isoforms varying in their glycan compositions (2,10). These compositions vary with respect to branching and terminal AMS residues which affect their physical-chemical properties. The biological effect of such spectra of isoforms, which gradually may vary in their half-life in the circulation and in their effects at the target organ, will be a resultant of that of the multiple isoforms (16,20,21). Therefore, in our physiological and clinical studies, we have chosen to express the number of AMS, SU, and SA per glycoform molecule or per glycan as the median value of that of the multiple isoforms.

The time-resolved sandwich fluoroimmunoassays used in these studies have several advantages. The background level is extremely low, and the method combines a high sensitivity with robustness and high reproducibility. As the reagents are added in large excess in sandwich techniques, the effect of differences in avidity between antibody and epitope on the hormone is small compared with that in competitive binding assays. The possibility to measure the hormones directly in the eluates from electrophoreses and gel filtrations is important and reduces the risk of selective loss of isoforms.

Circulating blood plasma contains fibrinogen, while our study was carried out on serum samples lacking fibrinogen. However, the described methods were not possible for plasma samples, regardless of anticoagulant, due to interferences of the fibrinogen molecule in the separation columns.

The use of the larger serum proteins for calibration of the 180 gel filtrations included in the study made it possible to estimate the size of the glycoforms with a high precision. The 30% level at calibration was chosen as it resulted in the best reproducibility compared with higher or lower levels. Also the electrophoreses were highly reproducible due to several calibration procedures. These include the addition of a lipid solution and serum albumin and measurements of their positions after electrophoresis. The calibration procedures also include carefully determined mobility of each hormone when AMS-free and the mobility increase per AMS on the molecules for FSH, LH, and TSH. The serum proteins added, both at gel filtration and electrophoresis, prevented trapping of analytes due to non-specific adsorption to plastic syringes and tubes and to glassware and possibly also to the Sephadex and agarose material.

The low-glycosylated FSH molecules found in pituitary and urinary extracts were first interpreted as di-glycosylated FSH molecules (13,14). Later it was shown that the majority of these glycoforms were tri-glycosylated with one glycan on the β-subunit (15,16,22). This was further supported in the present study, as the number of AMS per glycan was similar on the low- and the fully glycosylated FSH when assuming three glycans on the low-glycosylated one. The ratio between the two mean values was 1.04. From this follows that the low-glycosylated FSH glycoforms in a previous publication on FSH glycoforms in sera obtained during the menstrual cycle were tri- and not di-glycosylated (17). Di-glycosylated FSH forms were not detected in serum samples in this study, but we have found these glycoforms in very low concentrations in pituitary extracts (unpublished observation).

The ratio values of percent SU out of total AMS per molecule on low- versus fully glycosylated glycoforms were used in the formula to calculate the number of SU and SA per glycoform molecule. These ratio values have to be established for the different groups of individuals investigated in physiological and clinical studies. The ratio values were above 1 in all the 72 estimations. From this follows that the ratio values of SU/SA residues were higher for the low-glycosylated than for the fully glycosylated FSH, LH, and TSH.

The hormones are secreted in a pulsatile manner, and the compositions of the isoforms continuously change after each pulse. The disappearance rate of FSH in the human circulation is mainly regulated by the terminal SA residues on the glycans which prolong the survival (11). The disappearance rate of the LH and TSH molecules is regulated both by the terminal SA and SU residues on the glycans. Molecules with two or more terminal SU residues are quickly removed from the human blood circulation suggesting a mannose/sulfonated N-acetylgalactosamine-specific receptor in the human liver similar to that in rodents (23,24).

The composition of isoforms of each glycoform detected in a serum sample represents the isoforms circulating at the moment the blood sample was taken. In physiological and clinical studies we assume that this composition is close to that reaching the hormone receptors at the target organ.

In conclusion, all examined healthy individuals and patients consistently displayed the following six glycoforms in the blood circulation: FSHtri, FSHtetra, LHdi, LHtri, TSHdi, and TSHtri. The relative frequencies of the two glycoforms of each hormone and their median numbers of AMS, SU, and SA residues per molecule may vary considerably due to sex, age, and different physiological and clinical situations. The methods developed in the present study can be used in physiological and clinical studies for better understanding of the biology and the significance of these variations.
